# Supplementary material for: Impact of the COVID-19 pandemic on the mental health of professionals in 77 hospitals in France
Source: PLoS One. 2022 Feb 16;17(2):e0263666. doi: 10.1371/journal.pone.0263666 (PMC8849482; doi:10.1371/journal.pone.0263666)
Supplement: S1 Table — (DOCX) [file pone.0263666.s001.docx]

**Impact of the COVID-19 pandemic on the mental health of professionals in 77 hospitals in France**

**A Fournier et al**

**Supplementary Material 1**

**Table 1. List of French Departments participating in the study (PsyCOVID all professionals – June-September 2020, France).**

| Departements | Number of professionals per category | | | Total number of professionals (%) |
| --- | --- | --- | --- | --- |
|  | Public | Privé | Autre |  |
| Ain | 82 | 0 | 0 | 82 (1.9) |
| Aisne | 18 | 0 | 0 | 18 (0.4) |
| Alpes-Maritimes | 7 | 14 | 1 | 22 (0.5) |
| Ardèche | 4 | 0 | 0 | 4 (0.1) |
| Ardennes | 87 | 0 | 1 | 88 (2) |
| Ariège | 1 | 0 | 0 | 1 (0) |
| Aube | 111 | 0 | 0 | 111 (2.5) |
| Aude | 4 | 0 | 0 | 4 (0.1) |
| Aveyron | 2 | 0 | 0 | 2 (0) |
| Bas-Rhin | 18 | 1 | 0 | 19 (0.4) |
| Bouches-du-Rhône | 1 | 0 | 0 | 1 (0) |
| Calvados | 34 | 0 | 0 | 34 (0.8) |
| Charente | 52 | 0 | 0 | 52 (1.2) |
| Charente-Maritime | 112 | 0 | 0 | 112 (2.6) |
| Cher | 5 | 0 | 1 | 6 (0.1) |
| Côte-d'Or | 462 | 1 | 0 | 463 (10.6) |
| Côtes-d'Armor | 15 | 0 | 0 | 15 (0.3) |
| Doubs | 13 | 0 | 3 | 16 (0.4) |
| Essonne | 6 | 72 | 0 | 78 (1.8) |
| Eure | 10 | 0 | 0 | 10 (0.2) |
| Eure-et-Loir | 66 | 0 | 1 | 67 (1.5) |
| Finistère | 39 | 0 | 0 | 39 (0.9) |
| Gard | 2 | 0 | 0 | 2 (0) |
| Gers | 15 | 0 | 0 | 15 (0.3) |
| Haut-Rhin | 103 | 1 | 0 | 104 (2.4) |
| Haute-Garonne | 2 | 0 | 0 | 2 (0) |
| Haute-Saône | 100 | 0 | 7 | 107 (2.4) |
| Haute-Savoie | 20 | 0 | 0 | 20 (0.5) |
| Hautes-Pyrénées | 3 | 0 | 0 | 3 (0.1) |
| Hauts-de-Seine | 74 | 1 | 0 | 75 (1.7) |
| Hérault | 16 | 0 | 0 | 16 (0.4) |
| Ille-et-Vilaine | 43 | 0 | 2 | 45 (1) |
| Indre | 6 | 0 | 0 | 6 (0.1) |
| Indre-et-Loire | 16 | 0 | 0 | 16 (0.4) |
| Isère | 129 | 0 | 2 | 131 (3) |
| Jura | 67 | 0 | 1 | 68 (1.6) |
| La Réunion | 2 | 0 | 0 | 2 (0) |
| Landes | 23 | 0 | 0 | 23 (0.5) |
| Loire | 95 | 1 | 0 | 96 (2.2) |
| Loire-Atlantique | 69 | 1 | 0 | 70 (1.6) |
| Loiret | 160 | 0 | 3 | 163 (3.7) |
| Lot | 5 | 0 | 0 | 5 (0.1) |
| Maine-et-Loire | 56 | 0 | 0 | 56 (1.3) |
| Marne | 1 | 0 | 0 | 1 (0) |
| Mayenne | 3 | 0 | 0 | 3 (0.1) |
| Meurthe-et-Moselle | 77 | 0 | 0 | 77 (1.8) |
| Meuse | 12 | 0 | 0 | 12 (0.3) |
| Morbihan | 59 | 0 | 0 | 59 (1.4) |
| Moselle | 85 | 0 | 0 | 85 (1.9) |
| Nord | 473 | 53 | 18 | 544 (12.4) |
| Oise | 17 | 0 | 0 | 17 (0.4) |
| Orne | 3 | 0 | 0 | 3 (0.1) |
| Paris | 21 | 0 | 0 | 21 (0.5) |
| Pas-de-Calais | 174 | 1 | 2 | 177 (4.1) |
| Pyrénées-Orientales | 22 | 0 | 0 | 22 (0.5) |
| Rhône | 16 | 1 | 1 | 18 (0.4) |
| Saône-et-Loire | 264 | 0 | 7 | 271 (6.2) |
| Sarthe | 64 | 0 | 0 | 64 (1.5) |
| Seine-et-Marne | 9 | 0 | 0 | 9 (0.2) |
| Seine-Maritime | 131 | 0 | 3 | 134 (3.1) |
| Seine-Saint-Denis | 12 | 0 | 0 | 12 (0.3) |
| Somme | 40 | 0 | 2 | 42 (1) |
| Tarn | 10 | 0 | 0 | 10 (0.2) |
| Tarn-et-Garonne | 4 | 0 | 0 | 4 (0.1) |
| Territoire de Belfort | 141 | 0 | 0 | 141 (3.2) |
| Val-d'Oise | 42 | 0 | 0 | 42 (1) |
| Val-de-Marne | 6 | 2 | 0 | 8 (0.2) |
| Var | 11 | 0 | 0 | 11 (0.3) |
| Vendée | 142 | 0 | 0 | 142 (3.2) |
| Vienne | 1 | 0 | 0 | 1 (0) |
| Vosges | 11 | 0 | 1 | 12 (0.3) |
| Yonne | 36 | 0 | 2 | 38 (0.9) |
| Yvelines | 121 | 0 | 0 | 121 (2.8) |
