# Supplementary material for: Impact of the COVID-19 pandemic on the mental health of professionals in 77 hospitals in France
Source: PLoS One. 2022 Feb 16;17(2):e0263666. doi: 10.1371/journal.pone.0263666 (PMC8849482; doi:10.1371/journal.pone.0263666)
Supplement: S2 Table — (DOCX) [file pone.0263666.s002.docx]

**Impact of the COVID-19 pandemic on the mental health of professionals in 77 hospitals in France**

**A Fournier et al**

**Supplementary Material 2**

**Table 2. Number of participants in each professional category having either GHQ-12 and/or IES-R scores above the threshold indicative of the presence of the disorder (PsyCOVID all professionals – June – September 2020, France).**

|  | GHQ-12≥3  &  IES-R>33 | GHQ-12≥3 | IES-R>33 |
| --- | --- | --- | --- |
| Radiology staff | 22 (33.85) | 36 (55.38) | 24 (36.36) |
| Quality/hygiene/security/environment | 43 (33.08) | 88 (67.69) | 47 (35.07) |
| Nurses’ aides | 96 (32.54) | 184 (62.37) | 108 (35.53) |
| Welcome desk/orientation | 57 (25.45) | 138 (61.61) | 60 (26.43) |
| Midwives | 18 (25) | 50 (69.44) | 18 (25) |
| Nurses | 211 (23.87) | 520 (58.82) | 228 (25.59) |
| Physiotherapists | 12 (20.69) | 38 (65.52) | 12 (20) |
| Nursing managers | 79 (20.41) | 238 (61.5) | 93 (23.72) |
| Social workers | 14 (18.67) | 46 (61.33) | 14 (18.42) |
| Procurement/logistics | 19 (18.45) | 56 (54.37) | 20 (19.42) |
| Administration | 91 (18.27) | 279 (56.02) | 94 (18.47) |
| Laboratory staff | 19 (16.81) | 55 (48.67) | 19 (16.81) |
| Maintenance workers, computer engineers | 15 (13.27) | 53 (46.9) | 15 (13.27) |
| Instructors/training staff | 7 (12.73) | 31 (56.36) | 8 (14.29) |
| Pharmacy workers | 13 (11.82) | 66 (60) | 18 (16.07) |
| Physicians | 80 (11.32) | 365 (51.63) | 89 (12.5) |
| Clinical research staff | 14 (11.2) | 59 (47.2) | 16 (12.8) |
| Psychologists | 20 (10.31) | 91 (46.91) | 21 (10.82) |

*Note.* n (%).
